# Differential impacts of hemin and free iron on amoxicillin susceptibility in *ex vivo* gut microbial communities

**DOI:** 10.3389/fmicb.2025.1629464

**Published:** 2025-12-01

**Authors:** Francesco Pagano, Devin H. Bemis, Rahiya Rehman, Jason M. Shapiro, Peter Belenky

**Affiliations:** 1 Department of Molecular Microbiology and Immunology, Brown University, Providence, RI, United States; 2 Division of Pediatric Gastroenterology, Hepatology and Nutrition, Saint Louis University School of Medicine, Saint Louis, MO, United States; 3 Division of Pediatric Gastroenterology, Nutrition and Liver Diseases, Hasbro Children’s Hospital, Providence, RI, United States

**Keywords:** antibiotic susceptibility, *ex vivo* gut microbiome culture, iron metabolism, microbial metabolism, metabolic modulation, beta lactam antibiotics, hemin, 16S rRNA sequencing

## Abstract

**Introduction:**

The rise of antibiotic-resistant infections worldwide has created a need to enhance the efficacy of existing antibiotics. Modification of metabolism has been shown to potentiate antibiotic lethality. In this study, we employed a novel ex vivo microbiome culture approach to study the effects of different forms of iron on amoxicillin susceptibility.

**Methods:**

Synthetic and human stool-derived microbiota were cultured and treated with amoxicillin, with growth monitored by optical density. These samples were sequenced using an Oxford nanopore long-read 16S rRNA V4–V9 approach and computationally defined using the Emu algorithm. The validity of this pipeline was confirmed with consortia, murine cecal content, and a human stool sample. The stool-derived community was then cultured for 24 h with ranging concentrations of either hemin, FeSO_4_, or FeCl_3_ and concurrent amoxicillin dosage, then profiled to identify the effects of different forms of iron on amoxicillin susceptibility.

**Results:**

Alpha diversity, beta diversity, and normalized relative abundances confirmed the efficacy of the selected *ex vivo* pipeline, allowing for ~77% species retention over 24 h. Treatment of communities with hemin protected *Bacteroides*, *Escherichia-Shigella*, *Parabacteroides*, and *Parasutterella* against amoxicillin, while two forms of free iron did not.

**Discussion:**

This *ex vivo* pipeline enables reproducible assessment of how metabolic modulators like hemin alter amoxicillin susceptibility, highlighting a link between iron-sequestering genera and antibiotic-protection. Future mechanistic insights may support hemin-based strategies to boost antibiotic efficacy.

## Introduction

The microbiome can be defined as the collection of all genetic material from microorganisms within a distinct environment. Due to compositional variations associated with age, genetics, geography, diet, and the use of antibiotics, metabolic function serves as a better proxy for microbiome health (Berg et al., 2020; Wen and Duffy, 2017; Rinninella et al., 2019). Disruption to the gut through the aforementioned factors can lead to dysbiosis. This state is characterized by the reduction of microbial diversity, expansion of Proteobacteria and reduction of Firmicutes, and is largely associated with disease progression (Weiss and Hennet, 2017; Hrncir, 2022). Antibiotics often lead to antibiotic-induced dysbiosis (AID), resulting in a drastic reduction in microbial diversity and bacterial load (Patangia et al., 2022). Changes in the microbiome in the short term can lead to acute complications such as diarrhea (Hrncir, 2022; Goldenberg et al., 2015) and increase the possibility of infection (Maciel-Fiuza et al., 2023).

Amoxicillin is a broad-spectrum antibiotic of the penicillin group and operates through targeting penicillin-binding proteins to inactivate and shut down synthesis of peptidoglycan, leading to lysis and cell death (Centers for Disease Control and Prevention, 2022; Huttner et al., 2020). Overprescription of antibiotics has led to a rise in bacterial strains that are resistant to frontline antibiotics. In 2019 alone, 4.95 million deaths occurred globally as a result of drug-resistant infections (Walsh et al., 2023). Patients are affected not only through increased risk of mortality and morbidity from direct infection but also from a change in medical practice that may result from an inability to utilize antibiotics either prophylactically or clinically in many routine medical procedures (Patangia et al., 2022; Walsh et al., 2023; Hutchings et al., 2019). We need reproducible, cost-effective tools to study and enhance antibiotic efficacy against resistant infection.

The development of *ex vivo* culturing methods has been relatively successful in creating samples that closely resemble host microbiomes. However, representative microbial communities are still compositionally different from their starting inoculum, limiting the translatability of findings (Hirano et al., 2023; Tao et al., 2023; Cheng et al., 2022; Goodman et al., 2011; Aranda-Díaz et al., 2022; Celis et al., 2023). Cultures often exhibit bias or overrepresentation of species that thrive on the specific nutrients of the selected media, since no media can support the growth of all microbial members equally (Średnicka et al., 2023). Modified Gifu Anaerobic Media (mGAM) has previously been used to culture a large number of microbes spanning various phyla, and has been used to establish stable synthetic communities (Garcia-Santamarina et al., 2024) and grow *ex vivo* samples from mice (Tao et al., 2023). mGAM uses a large ratio of fibers to simple sugars, digested proteins, host factors and hydrogen acceptors through sodium thioglycolate and L-cysteine to support the growth of anaerobic microbes (van Bergen et al., 2016; Lobo et al., 2018). The supplementation of mGAM with a secondary media can be used to support the growth of other microbes that may not otherwise be supported.

Recent studies have identified that modifying the metabolic flux of bacteria can drastically affect the killing efficacy of antibiotics (Walsh et al., 2023). Early works identified that genetically modifying *E. coli* to increase their aerobic respiration led to an increase in antibiotic susceptibility to ampicillin (Walsh et al., 2023; Lobritz et al., 2015); this phenomenon may be partly due to an increase in oxidative stress (Belenky et al., 2015). This observation is further supported with the observation that *B. thetaiotaomicron* blooms in response to amoxicillin and upregulates polysaccharide utilization (Cabral et al., 2019). Both *in vitro* and *in vivo, B. thetaiotaomicron* displays polysaccharide-mediated tolerance (PM-tolerance), gaining protection from amoxicillin when metabolizing pectin, while glucose exposure increases susceptibility. This heightened sensitivity is linked to a surge in antibiotic-triggered ATP production and activation of a shortened electron transport chain (Cabral et al., 2019; Nilson et al., 2024). In more complex communities *in vivo*, high fiber diets reduced dysbiosis as a result of amoxicillin treatment compared to standard diets, and glucose exacerbated antibiotic-induced dysbiosis through blooms in Proteobacteria (Penumutchu et al., 2023). Metabolically, it was identified that glucose increased oxidative metabolism while fiber repressed oxidative metabolism (Penumutchu et al., 2023). Work conducted in *E. coli* indicates that the beta-lactam-induced metabolic burden results from a futile cycle of peptidoglycan synthesis to degradation, and that removing this activity through direct inhibition or the reduction of ATP synthesis results in reduced toxicity (Lobritz et al., 2015; Belenky et al., 2015; Cho et al., 2014). As such, overloading the electron transport chain to increase ATP production may potentiate the response of microbes in complex communities to antibiotics.

One way to modify microbial ATP production is through the supplementation of iron to microbial communities. Iron is an essential metal for most living organisms. Iron can exist in a ferric (Fe^3+^) or ferrous (Fe^2+^) form, and can be included in cofactors involved in various biological processes (Py and Barras, 2010; Sawicki et al., 2015). In aerobic conditions, iron can produce hydroxyl radicals through reactions with hydrogen peroxide to potentiate antibiotic susceptibility (Ranji-Burachaloo et al., 2018; Cadet et al., 2010; Sheng et al., 2015; Rachmilovich-Calis et al., 2009). The Fenton reaction can also occur anaerobically at lower efficiency (Merino et al., 2020), and iron reactions have been shown to be able to participate in nitrate reduction through iron oxidation in anoxic conditions (Liu et al., 2019). Anaerobically, ferric iron can act as a terminal electron acceptor in the electron transport chain to help produce ATP (Kashefi et al., 2002; Nealson and Saffarini, 1994). Studying the role iron has on antibiotic efficacy is important, as approximately 25% of the world population suffers from some form of anemia (Hess et al., 2023).

The role iron may have in antibiotic susceptibility has been explored, though results are conflicting. The reduction of iron from media has been found to cause a decrease in *E. coli*’s resistance to clindamycin, mupirocin, tetracycline, and clarithromycin, while approaches using chelators to reduce iron concentrations found that decreasing iron led to an increase in resistance to cephalosporin, ampicillin, chloramphenicol, methicillin, and vancomycin while having no effect on susceptibility to erythromycin, spectinomycin, chloramphenicol, rifampicin, and tetracycline (Ezraty and Barras, 2016; Mochizuki et al., 1988; Kohanski et al., 2007; Wang and Zhao, 2009; Liu and Imlay, 2013; Ma et al., 2015). Other species such as *P. aeruginosa* show similar conflicting results. The addition of iron to media caused reduced resistance to ampicillin, norfloxacin, gentamicin, ofloxacin, and cefsulodin; the opposite was also observed, however, where addition of iron has also caused increased resistance to tobramycin and tigecycline (Ezraty and Barras, 2016; Yeom et al., 2010; Kreamer Naomi et al., 2015; Oglesby-Sherrouse et al., 2014). In complex communities *in vivo*, oral supplementation with iron sulfate through diet post-antibiotic exposure lead to an initial decrease in alpha diversity and has been found to lead to an increase in *Parasutterella* and *Bacteroides* genera (Cuisiniere et al., 2021). The impact that iron trapped in cofactors, such as in hemin, has on the effectiveness of antibiotics on complex microbial communities has yet to be elucidated. Hemin alone has previously been shown to shift murine ileal microbiota composition *in vivo*, as well as human microbiota *in vitro* (Celis et al., 2023; Li et al., 2024).

In this study, we propose a novel culturing pipeline that allows us to monitor the role various forms of iron have on amoxicillin susceptibility in complex communities *ex vivo.* The Nanopore MinION enables amplification and sequencing of longer 16S rRNA regions, improving taxonomic resolution when paired with error-correction tools such as Emu that minimize biases in abundance estimates (Tyler et al., 2018; Szoboszlay et al., 2023; Workman et al., 2019; Curry et al., 2022). The relationship iron in its ferric form, ferrous form, and as part of a cofactor with amoxicillin was evaluated through this pipeline, allowing for the identification of four unique genera protected against amoxicillin killing in response to increased hemin, and not free iron, in media.

## Methods

### Media preparation

Minimal Gifu Anaerobic Broth Media (mGAM) (Hyserve 1,005,433) was prepared per manufacturing instructions; briefly, 41.7 g was suspended per liter of H_2_O and heated to allow to dissolve before autoclaving at 121°C for 15 min. All mGAM was supplemented with 1 mg/mL of mucin (Sigma-Aldrich M1778) prior to autoclaving to allow for dissolving. Media was placed in anaerobic chamber at least 24 h in advance to experimentation to allow for exchange of gasses. Desulfovibrio Postgate Medium (DPM) was prepared using a modified version of a DSMZ established protocol (DSMZ, n.d.); briefly, Solution A (0.5 g/L K_2_HPO_4_, 1.0 g/L NH_4_Cl, 1.0 g Na_2_SO4, 0.1 g CaCl_2_ × 2H_2_O, 2.0 g MgSO_4_ × 7H_2_O, 2.0 g Na-L-lactate, 1.0 g Yeast extract, 980 mL H_2_O), Solution B (0.5 g FeSO_4_ × 7H_2_O, 10 mL H_2_O), and Solution C (0.1 g Sodium thioglycolate, 0.1 g Ascorbic acid, 10 mL H_2_O), were prepared while spinning. Solution A was filter sterilized using a 0.22 μm bottle vacuum filter (Corning 430,015) using sterile flame technique followed by autoclaving at 121°C for 15 min, and Solutions B and C were filter sterilized using 0.22 μm centrifuge tube filters (Millipore SCGP00525) by sterile flame technique. Solutions B and C were added to room temperature Solution A and immediately placed in the anaerobic chamber to reduce. Working media for culturing (10%DPM/mGAM+mucin) was created by diluting a ratio of 1 mL DPM in 9 mL of mGAM + 1 mg/mL mucin using sterile flame techniques.

### Anaerobic chamber conditions

A Coy Lab Products vinyl anaerobic chamber (Coy Labs 7,150,000) was used to create anaerobic conditions for all cultures and culturing conditions. The vinyl anaerobic chamber is outfitted with an incubator (Coy Labs 6,100,000) set at 37°C, a recirculating HEPA atmospheric filtration system (Coy Labs 8,537,025 V), an anaerobic gas infuser (Coy Labs 8,100,110) and a dehumidifier (8,533,110) to ensure anaerobic conditions. The chamber is also outfitted with an anaerobic monitor, model 12 (Coy Labs 6,250,000) to allow for monitoring of anaerobic conditions and hydrogen % content. Atmospheric makeup of the machine is created using a mixture of two gasses: pure nitrogen gas, and a mixed gas containing 5% CO_2_, 5% H_2_, and balance N_2_. Gasses are mixed into the chamber to obtain a 2.0–2.5% hydrogen content, and an O_2_ = 0–10 ppm.

### Establishment of consortia

Eight bacterial species were chosen to create a synthetic community: *Akkermansia muciniphila, Bacteroides fragilis, Bacteroides thetaiotaomicron, Bifidobacterium longum, Desulfovibrio desulfuricans, Escherichia coli, Lactobacillus johnsonii,* and *Roseburia hominis.* Supplementary Table S1 includes specific vendor information for bacterial strains purchased. All species, except for *D. desulfuricans* and *B. longum*, were grown in 5 mL mGAM +1 mg/mL mucin media for 24 h, with the two exceptions being grown in 5 mL DPM for 48 h. Then 150 μL of each culture was inoculated into a plate and diluted with equal parts sterile filtered PBS. OD_600_ measurements were taken using the Molecular Devices SpectraMax M3 Spectrophotometer using SoftMax Pro v6, blanking the culture to its original medium. Cultures were then consolidated in the following ratios in 10% DPM/mGAM + 1 mg/mL mucin to achieve an OD_600_ of 0.1: 1 part each *B. thetaiotaomicron, B. fragilis, E. coli,* and *B. longum* to 5 parts each *R. hominis, L. johnsonii, D. desulfuricans,* and *B. longum*.

### Collection of mouse cecal content into chamber

Cecums from male C57BL/6 J mice (Charles River) were collected per IACUC protocol 23-05-0013. Mice were then dissected, and the entire intestinal tract was collected into a sterile plate and transferred to the anaerobic chamber. In the anaerobic chamber, the cecum was isolated from the rest of the GI tract and its contents (approximately 200 μL) were transferred into approximately 15 mL of 10% DPM/mGAM + 1 mg/mL mucin media and cultured for approximately 2 h.

### Human material processing

A human stool sample was obtained through a collaboration between Brown University and Hasbro Children’s Hospital (IRB 1930822-3) by Rahiya Rehman, and immediately frozen at −80°C. Approximately 130 mg of stool was weighed out and placed into 20 mL of 10% DPM/mGAM + 1 mg/mL mucin, pipetted to break up the matter, and grown for 24 h while shaking at ~400 rpm. Cultured content was then frozen at −80°C in 1 mL aliquots in 20% filtered glycerol (Fisher Sci G331) until the start of the experiment. On the day of the experiment, frozen cultures were diluted 1:200 in media prior to the start of the experiment (this sample is used for Supplementary Figure S1 only).

For cultures treated with iron and amoxicillin, a human stool sample was obtained through BioIVT (BioIVT HUMANFECES-0000263); donor information was deidentified prior to receipt of material. Upon arrival, the sample was immediately frozen at −80°C. Approximately 130 mg of stool samples were weighed out and placed into 20 mL of 10% DPM/mGAM + 1 mg/mL mucin, pipetted to break up matter, and grown until exponential growth phase (~0.2) for 5 h while shaking at ~400 rpm. Content was then frozen at −80° C in 1 mL aliquots in 20% filtered glycerol (Fisher Sci G331) until the start of the experiment. On the day of the experiment, frozen culture content—originating from the single donor—is diluted 1:200 in media and allowed to grow until once again at the exponential growth phase of approximately 0.2 (~6 h) before treatment (this sample is used for experimental data shown in Figures 2, 3).

### *Ex vivo* culturing of microbial samples

Input cultures, prior to treatment, were first grown to an optical density of approximately 0.2. Cultures were then treated with hemin (Fisher Sci AAA1116503), FeSO_4_ (Sigma-Aldrich F8633) or FeCl_3_ (Fisher Sci AC169430050), and then 300 μL was added per well to a 96 well flat bottom plate (Corning 3,370) and treated with amoxicillin (Millipore A8523) at concentrations of 50 μg/mL, 10 μg/mL, 12.5 μg/mL, 3.125 μg/mL, 2 μg/mL, or 0 μg/mL using amoxicillin stocks diluted in DMSO (Fisher Sci BP2311). Cultures’ optical densities were monitored for 24 h using the Cerillo Alto Kinetic Microplate Reader while incubating at 37°C in the anaerobic chamber, and cultures were collected at 24 h post treatment for downstream applications.

### DNA cleanup

Cultures collected post treatment were processed via a selected portion of the Zymo Research HostZERO Microbial DNA kit (Zymo D4310). Briefly, 300 μL of sample was centrifuged at 2,250 × g for 8 min to pellet solids, then the supernatant was removed. One microliter of Microbial Selection Enzyme (Zymo D4310350) in 100 uL of Microbial Selection Buffer (Zymo D431025) was added to the pellet, vortexed, and incubated at 37°C for 15 min. Samples were then treated with 20 uL of Proteinase K (D30012125, reconstituted at 20 mg/mL), vortexed, incubated at 55°C for 10 min, then diluted 1:1 in DNA/RNA Shield (R1100250, 1X Solution) and frozen at −80°C until further use.

### *Escherichia coli* Nissle spike-in and DNA cleanup test on synthetic community

*E. coli* Nissle 1917 (see Supplementary Table S1 for more information) was grown to stationary phase in mGAM + 1 mg/mL mucin (OD ~ 3–4) then diluted to an OD_600_ of 0.5. These *E. coli* cultures were then treated for 30 min with amoxicillin (Millipore A8523) at a concentration of 100 μg/mL and then mixed 1:1 into the synthetic community previously described, also mixed to a final OD_600_ of 0.5. These spiked communities were then treated with or without the DNA Cleanup step and had their 16S rRNA gene amplified and sequenced as described below.

### 16S rRNA amplification prep and sequencing

DNA for all samples was extracted using the ZymoBIOMICS Quick-DNA Fecal/Soil Microbe 96 Kit (Zymo D8011) following the manufacturer’s protocol. Total DNA was eluted into nuclease-free water and quantified using the dsDNA-BR kit on a Qubit 4.0 fluorometer (ThermoFisher Scientific, Waltham, MA, United States) before library preparation.

16S rRNA V4-V9 hypervariable regions were amplified from total DNA using barcoded 515F forward primers from the Earth Microbiome Project (Thompson et al., 2017) and using a matched set of reverse barcodes alongside the 1492R primer. See Supplementary Table S2 for reverse barcoded primer sequences used. Amplicons were created using 5X Phusion HF DNA Polymerase (Fisher Sci F530L) under the following PCR conditions: an initial denaturation at 98° C for 30 s followed by 25 cycles of 98°C for 10s, 57°C for 30 s, and 72°C for 30 s. Amplicons then underwent a final extension at 72°C for 5 min. Amplicons were visualized for confirmation of successful amplification via gel electrophoresis, and pooled at equal DNA weight before PCR cleanup using the Macherey-Nagel Gel and PCR Clean-Up kit (Macherey-Nagel 740609.250) following the manufacturer’s protocol. DNA libraries were prepared with Oxford Nanopore Technologies’ Native Barcoding 24 v14 kit (ONT SQK-NBD114-96), then sequenced with a MinION MK1B (ONT MIN-101B).

### Data analysis

16S rRNA sequences were basecalled using Nanopore’s Guppy Software v6.5.7 through the guppy_basecaller function. Reads were then demultiplexed using Nanopore’s Guppy Software v6.5.7 using the guppy_barcoder function, and barcodes and adapters were trimmed. Primary demultiplexed reads were then secondarily demultiplexed using Porechop with custom barcode inputs denoted from the Earth Microbiome Project (Thompson et al., 2017) and Supplementary Table S2. Taxonomic assignments were performed using the SILVA database (Quast et al., 2013) with Emu (Curry et al., 2022). Shannon Diversity index values and Bray–Curtis dissimilarity values were calculated using the phyloseq package v1.46.0 (McMurdie and Holmes, 2013) in RStudio v12.1 (McMurdie and Holmes, 2013; Lozupone and Knight, 2005).

### MIC determination

Bacterial species denoted in Supplementary Table S1 were grown anaerobically in mGAM + 1 mg/mL mucin for 24 h prior to the experiment. Experimental 96-well flat bottom plates (Corning 3,370) were setup with dilutions of amoxicillin (Millipore A8523) ranging in concentration from 0 to 250 μg/mL (final concentrations). Bacterial cultures were then diluted 1:1000 into media containing no iron, or media with supplementations of different concentrations of hemin (Fisher Sci AAA1116503), FeSO_4_ (Sigma-Aldrich F8633) or FeCl_3_ (Fisher Sci AC169430050), and 150 uL was then inoculated per well into the plates. Plates were then incubated at 37°C for 20 h before diluting 1:1 with 1X PBS (Fisher Sci BP39920) and OD_600_ measurements were taken using the Molecular Devices SpectraMax M3 Spectrophotometer using SoftMax Pro v6. Percent growth was then calculated against 0 μg/mL treated cultures, and the Gompertz Equation for MIC Determination was used to determine MIC_90_ with Prism GraphPad v10.4.1.

### Statistical analyses and visualizations

Specific details pertaining to statistical analyses for all experiments are detailed in the figure legends and results section. MaAsLin2 (Mallick et al., 2021) was used to analyze outputs from Emu. T-tests and Gompertz Equation for MIC Determination were performed in Prism GraphPad v10.4.1. All other graphs were created using Prism GraphPad v10.4.1.

## Results

### An *ex vivo* culturing approach allows for the successful cultivation and sequencing of complex microbial communities over 48 h

**Figure 1:**
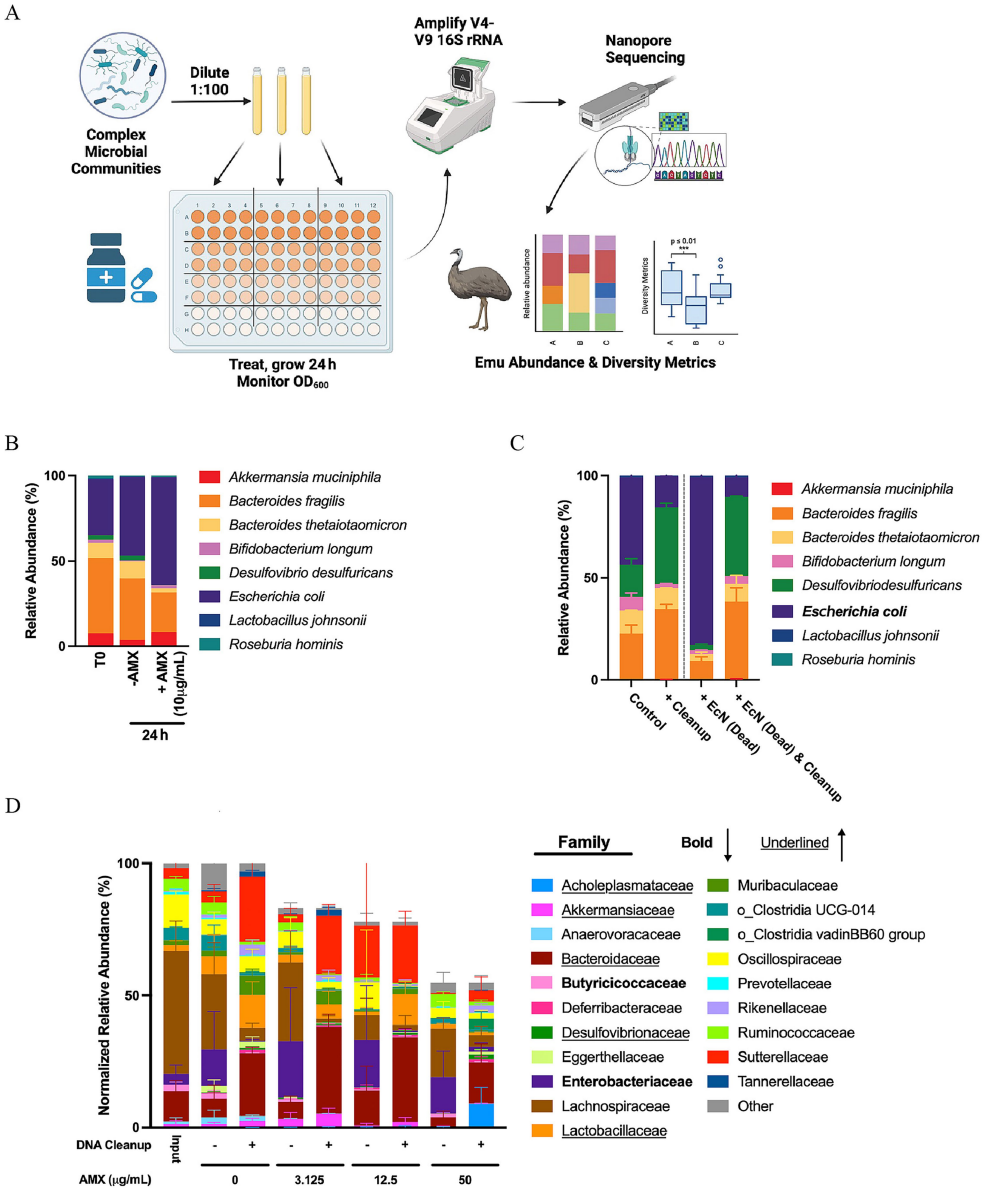
Establishment of pipeline in synthetic consortia community and murine cecal communities. **(A)** Experimental design for culturing various community inputs to obtain sequencing data. Figure was created using Biorender. **(B)** Average relative abundances of species comprising a synthetic community treated with or without amoxicillin over 24 h. Data are represented as average relative abundance for *n* = 3–5 replicates. **(C)** Average relative abundance of species of the synthetic community spiked with *E. coli* killed from 100 μg/mL AMX and treated with DNA Cleanup step. Data are represented as average relative abundance ± STD for *n* = 4. **(D)** Average normalized relative abundances of families within cecal communities treated with or without amoxicillin and/or the DNA cleanup step for 24 h. Families in bold represent families that are reduced in response to the cleanup step, while families underlined increase in response to the cleanup step. Data are represented as average relative abundance normalized against the highest OD_600_ ± STD for *n* = 6.

Though *ex vivo* culturing approaches have been used to identify changes in microbiomes (Hirano et al., 2023; Tao et al., 2023; Cheng et al., 2022; Goodman et al., 2011), large passage numbers of the communities meant to stabilize the microbiomes cause the passaged communities to differ substantially from original community inputs (Aranda-Díaz et al., 2022; Celis et al., 2023); a protocol is thus necessary for cultured communities to better resemble the original communities in question. A brief overview of the protocol developed is seen in Figure 1A. Communities, ranging from known synthetic communities to complex unknown human stool microbiome samples, were cultured out and treated with amoxicillin, with their optical density monitored. The cultured samples then have their 16S rRNA V4-V9 region amplified using a double barcoding approach modified from the Earth Microbiome Project (Thompson et al., 2017) and sequenced on the Nanopore MinION, then demultiplexed and aligned using the Emu algorithm (Curry et al., 2022). The Emu taxonomic alignment algorithm more accurately discerns species taxonomic information and overcome any potential barcode leakage previously seen with Nanopore sequencing (Bemis et al., 2025).

To test the validity of this pipeline, we cultured a synthetic community containing eight microbial species combined to act as the initial community input for the pipeline: *A. muciniphila, B. fragilis, B. thetaiotaomicron, B. longum, D. desulfuricans, E. coli, L. johnsonii,* and *R. hominis*. These microbes were chosen to represent a wide range of phyla, and more information on these species can be found in Supplementary Table S1. When this community was treated with 10 μg/mL amoxicillin for 24 h, there was a noticed increase in the relative abundance of *E. coli* in the community (Figure 1B). Because of the treatment of this contained community for 24 h with a bactericidal drug, it was possible that DNA from dead microbes, including *E. coli* could be represented downstream in the taxonomic abundances. To reduce the impact of DNA from killed microbes, we chose to use a DNA cleanup step derived from Zymo Research’s Host-Zero Microbial DNA Kit, comprising of a DNase treatment followed by a Proteinase K treatment prior to DNA extraction (Heravi et al., 2020). To test this step, we spiked our synthetic community with an equal OD_600_ of *E. coli* killed with 100 μg/mL of amoxicillin for 1 h and treated with or without the DNA cleanup steps to eliminate free floating DNA (Figure 1C). Treating the community with the DNA Cleanup alone caused a limited shift in the relative abundance of the community and a general reduction in the relative abundance of *E. coli*. When killed *E. coli* was spiked into a base community containing viable *E. coli*, the relative abundance of *E. coli* dominated as expected. Performing a cleanup on this “spike-in” community reduced the abundance of *E. coli* to mirror the community that had not been spiked with killed *E. coli,* indicating that the proposed cleanup was effective at eliminating killed bacteria.

While this cleanup step worked well for a known synthetic community, we still needed to confirm its effectiveness for mammalian samples. Additionally, we needed a way to compare relative abundance with the overall growth of the culture. In order to accomplish this, murine cecal samples were collected as the complex community input detailed in Figure 1A and treated with different amoxicillin concentrations for 24 h followed by the presence/absence of our DNA Cleanup step prior to amplification and sequencing (Figure 1D). Data generated from the pipeline was normalized to OD_600_ to integrate overall community growth with the relative abundance data. Treatment of the communities with amoxicillin created a stepwise reduction in total normalized relative abundance of the murine cecal samples. The implementation of the DNA cleanup step showed changes in a few families of bacteria within the communities. Families in bold reduce in normalized relative abundance as a result of the DNA cleanup, indicating that these families are overrepresented and experience more drastic killing as a result of the culturing and antibiotic treatment; these families identified include *Butyricicoccaceae* and *Enterobacteriaceae*. Families underlined indicate an increase in normalized relative abundance as a result of the DNA cleanup, indicating that these families are normally underrepresented without it; these families include *Acholeplasmataceae*, *Akkermansiaceae*, *Bacteroidaceae*, *Desulfovibrionaceae*, and *Lactobacillaceae*.

The pipeline was also tested using a patient stool sample that was cultured and frozen in 20% glycerol to act as starting communities in the pipeline. These samples retained their Shannon diversity over a 48 h culturing period and showed minimal variance and dissimilarity through Bray-Curtis beta-diversity when compared to the donor sample (Supplementary Figures S1A,B). Profiling of these samples in Supplementary Figure S1C showed that, although there were some shifts in families over time such *Bifidobacteriaceae*, *Sutterellaceae*, and *Tannerellaceae*, and *Muribaculaceae*, these shifts were not able to alter overall alpha and beta diversity form our donor sample. In order to identify species retention over time of these samples, a “Percent of Species Still Present from Baseline” value was calculated (Supplementary Figure S1D), where the percentage of species that retained at least 1% of their relative abundance values compared to the donor stool sample was identified. Culturing of the stool sample for 24 h allowed for a 1% species retention threshold of about 77%, while culturing the communities further out to 48 h resulted in a 1% species retention threshold of 43.25%. As populations reach their maximum OD600 over the course of the 48 h, the number of viable bacteria growing in culture was likely reduced due to toxicity of stationary phase cultures. This data particularly highlights the effectiveness of the culturing portion of the pipeline to retain species over time; it should be noted that increasing total culturing times begins to cause a degradation in the total number of species represented, so special care should be taken to reduce total culturing time.

### Hemin protects four genera against amoxicillin-induced death

Iron is a critical factor in bacterial metabolism, and previous research has shown that it may influence antibiotic efficacy (Ezraty and Barras, 2016; Cuisiniere et al., 2021). Given that human-derived components are a major source of iron in mGAM media, we selected hemin as a potential modulator of antibiotic lethality. Since iron can serve as a terminal electron acceptor in metabolic processes and anaerobic respiration (Nealson and Saffarini, 1994; Richter et al., 2012), we hypothesized that altering hemin concentrations within the media could push microbial communities toward a more energy-productive state, thereby enhancing antibiotic lethality. To test this hypothesis, we used a human microbiome biospecimen obtained from BioIVT as the input community.

**Figure 2:**
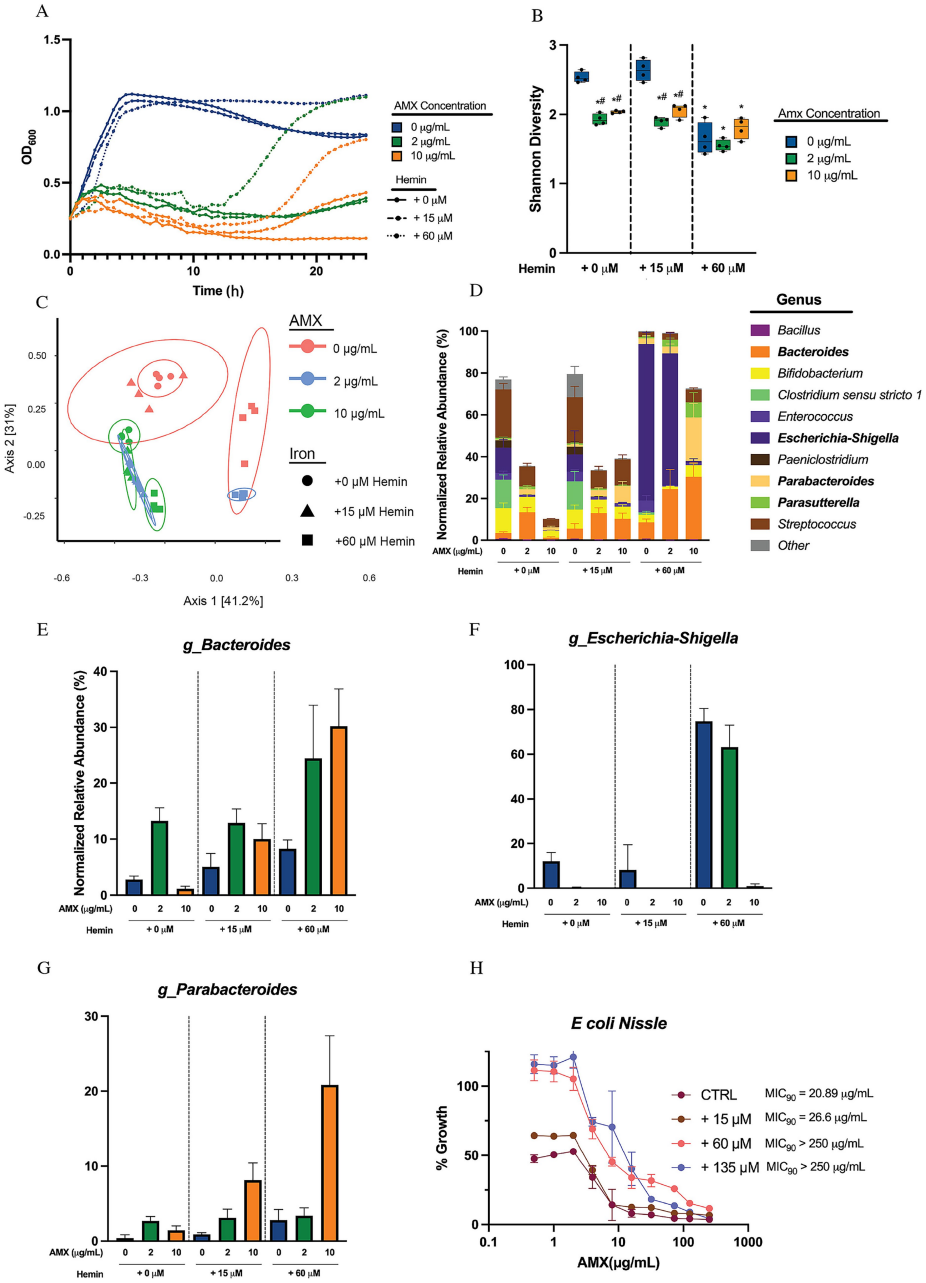
Hemin supplementation in media provides protection against amoxicillin susceptibility for four genera in human microbial sample cultures. **(A)** Optical density at 600 nm of microbial human stool sample cultures measured every 30 min over 24 h. Data are represented as means of OD_600_ of *n* = 6. **(B)** Alpha diversity of sample cultures as measured by Shannon diversity index. Black lines indicate the mean, with whiskers identifying minimum and maximums of the data for *n* = 4 (*p < 0.05, compared to 0 μg/mL AMX in + 0 μM hemin concentration; ^#^*p* < 0.05, compared to 0 μg/mL AMX in same hemin conditions). **(C)** PCoA of beta diversity of samples via Bray–Curtis Dissimilarity. **(D)** Average normalized relative abundances of genera. Top 23 genera are shown, with all other genera collapsed to “Other.” Genera in bold showed significance through MaAsLin2 due to the combinational effects of hemin and amoxicillin (FDR < 0.05). Data are represented as average relative abundance normalized against highest OD_600_ of experiment ± STD for *n* = 4. **(E)** Average normalized relative abundance of *Bacteroides* genus extrapolated from Figure 3D. Data are represented as average relative abundance normalized against highest OD_600_ of experiment ± STD for *n* = 4. **(F)** Average normalized relative abundance of *Escherichia-Shigella* genus extrapolated from Figure 3D. Data are represented as average relative abundance normalized against highest OD_600_ of experiment ± STD for *n* = 4. **(G)** Average normalized relative abundance of *Parabacteroides* genus extrapolated from Figure 3D. Data are represented as average relative abundance normalized against highest OD_600_ of experiment ± STD for *n* = 4. **(H)** Percent growth curve of *E. coli Nissle* treated with amoxicillin and hemin. MIC_90_ calculated using Gompertz equation for MIC determination where % growth = 10%. Data represented as average percent growth ± STD for *n* = 3–6.

We found that hemin supplementation alone slightly reduced the initial growth rate of the microbiome culture but ultimately resulted in a higher final OD_600_, seen in Figure 2A. When combined with 2 μg/mL or 10 μg/mL amoxicillin, hemin had a modest effect on early culture inhibition kinetics but led to higher final ODs compared to antibiotic treatment alone. This suggests that hemin facilitates microbiome recovery following initial AMX inhibition, potentially aiding post-antibiotic microbiome restoration or supporting bacterial survival in the presence of antibiotics.

We sequenced this community and first profiled alpha diversity at 24 h, finding that AMX led to the expected significant drop in Shannon diversity in both the + 0 μM and + 15 μM hemin groups (^#^*p* < 0.05). On the other hand, the + 60 μM hemin cultures started with a significantly lower Shannon diversity (*p < 0.05) without amoxicillin treatment compared to the + 0 μM hemin group and exhibited no significant changes in Shannon diversity as a result of amoxicillin treatments (Figure 2B). The Bray–Curtis dissimilarity beta diversity plot of the samples showed distinct concentration-driven separation along PC2 for amoxicillin either at zero or 15 μM hemin (Figure 2C). The presence of 60 μM hemin shifted the community into the positive PC1 quadrant. The low amoxicillin concentration was also shifted into that quadrant, while the high AMX concentration returned the hemin community to the zero-iron AMX-treated group.

**Figure 3:**
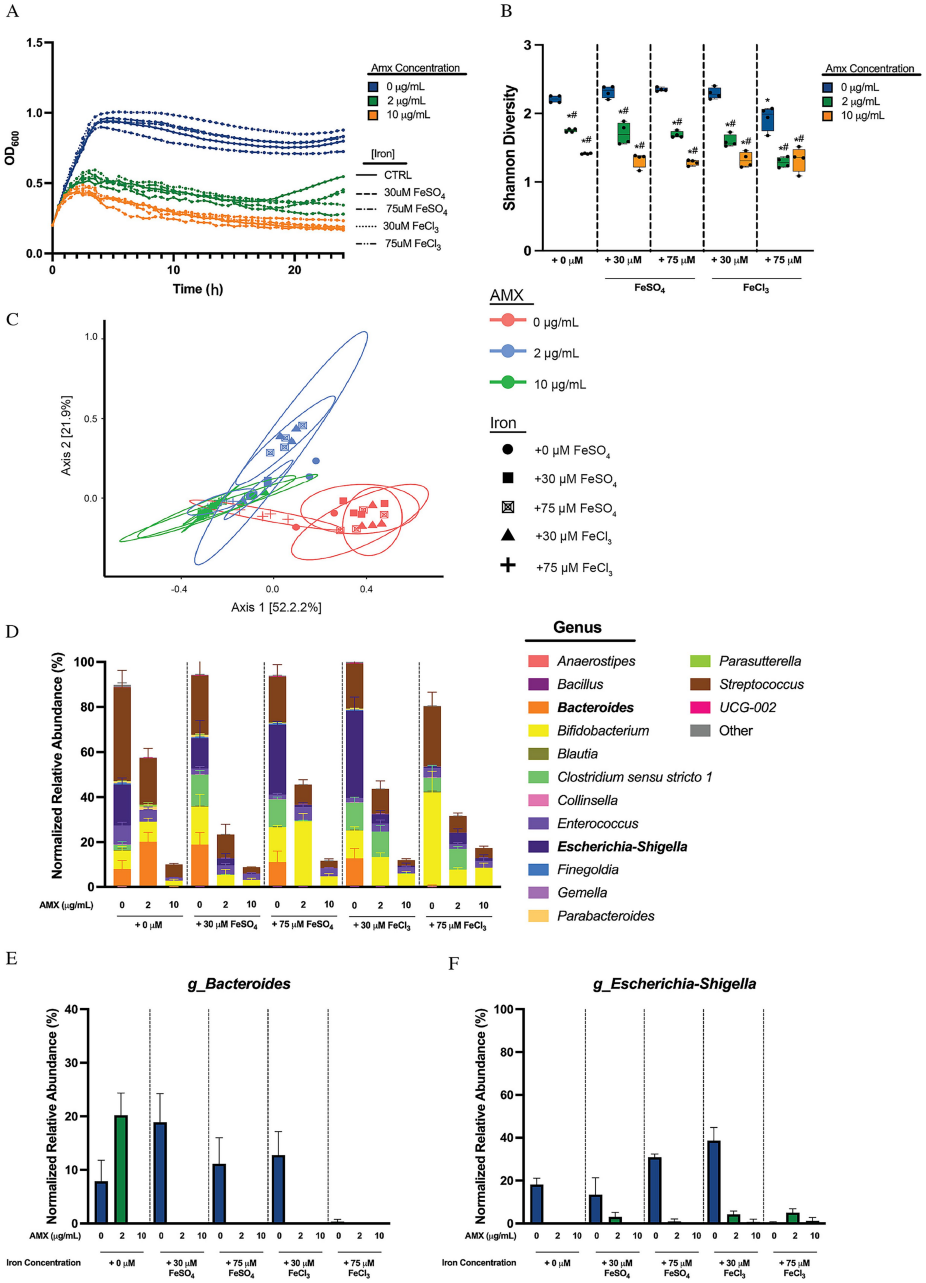
Free ferric and ferrous iron minimally impacts human microbial sample community makeup. **(A)** Optical density at 600 nm of microbial human stool sample cultures measured every 30 min over 24 h. Data are represented as means of OD_600_ of *n* = 6. **(B)** Alpha diversity of sample cultures as measured by Shannon diversity index. Black lines indicate the mean, with whiskers identifying minimum and maximums of the data for *n* = 4 (*p < 0.05, compared to 0 μg/mL AMX in + 0 μM free iron concentration; ^#^*p* < 0.05, compared to 0 μg/mL AMX in same free iron conditions). **(C)** PCoA of beta diversity of samples via Bray–Curtis Dissimilarity. **(D)** Average normalized relative abundances of genera. Top 15 genera are shown, with all other genera collapsed to “Other.” Genera in bold showed reduction to abundances in response to amoxicillin when given free iron. Data are represented as average relative abundance normalized against highest OD_600_ of experiment ± STD for *n* = 4. **(E)** Average normalized relative abundance of *Bacteroides* genus extrapolated from **(D)**. Data are represented as average relative abundance normalized against highest OD_600_ of experiment ± STD for *n* = 4. **(F)** Average normalized relative abundance of *Escherichia-Shigella* genus extrapolated from **(D)**. Data are represented as average relative abundance normalized against highest OD_600_ of experiment ± STD for *n* = 4.

We next examined the OD-normalized relative abundance of the communities and observed shifts in four major genera in response to the combined effects of increased hemin and amoxicillin: *Bacteroides, Escherichia-Shigella, Parabacteroides,* and *Parasutterella*, shown in bold in the Legend (Figure 2D). The significance of these changes was confirmed through MaAsLin2, and independently graphed and shown in Figures 2E–G and Supplementary Figure S2A. The *Bacteroides* genus, without additional hemin, initially bloom in low amoxicillin but are eventually reduced at higher amoxicillin concentrations. The addition of + 15 μM hemin changes this dynamic, stabilizing the relative abundance numbers for the group when treated with antibiotics. When given + 60 μM hemin, *Bacteroides* are further stabilized, and even bloom with AMX (Figures 2D,E). We tested the MIC of two representative species of this genus, *B fragilis* and *B thetaiotaomicron*. The addition of increasing amounts of hemin to culture media did not significantly shift the MIC_90_ of either of these species (Supplementary Figures S2C,D). It should be noted that the *Bacteroides* genus shifting in Figure 3D is comprised almost exclusively of an undetermined species of *Bacteroides*, and this phenomenon originally observed may be unique to this species.

In the presence of amoxicillin, *Escherichia-Shigella* does not expand until supplemented with + 60 μM hemin, allowing for a shift in overall resistance to amoxicillin and allowing for major growth in 2 μg/mL of amoxicillin. High hemin concentrations also support a large bloom in normalized relative abundance of *Escherichia-Shigella* without the presence of AMX (Figures 2D,F). We also found with *E. coli* Nissle 1917 that hemin supplementation increased the MIC_90_ at least 10-fold (Figure 2H). It should be noted that this strain of *E. coli* is not the same strain seen in our dataset. *Parabacteroides* and *Parasutterella* both exhibited a bloom in the presence of amoxicillin and high hemin concentrations in the media (Figures 2D,G; Supplementary Figure S2A) indicating that hemin may have a protective effect for these genera against amoxicillin.

The genus *Enterococcus* showed no significant change to amoxicillin susceptibility in response to increasing amoxicillin but did show interesting nonsignificant trends, leading us to the impact of hemin on the MIC_90_ of an *E. faecalis* strain. We found that giving this strain hemin at any concentrations led to a drastic drop in the MIC_90_ to about 1/10 of control conditions (Supplementary Figures S2B,E), indicating that hemin drastically sensitized this strain of *E. faecalis* to antibiotic killing.

### Free, soluble iron provides minimal impact on microbial community makeup

Iron can exist in many forms, both trapped in molecules like hemin (chloroprotoporphyrinIX iron(III)), or in soluble forms in both ferric (Fe^3+^) and ferrous (Fe^2+^) iron. To test whether the phenomena identified with hemin was conserved among iron species or unique compared to free iron sources, the communities were grown and monitored for 24 h in the presence of either Fe(II)SO_4_ or Fe(III)Cl_3_. The presence of free iron, either ferrous (II) or ferric (III), did not affect overall optical density of the cultures (Figure 3A). This holds true between amoxicillin treated groups, where it is shown that amoxicillin causes a stepwise reduction in OD_600_ but this drop is not influenced by the presence of free iron. The 24 h Shannon diversity showed a drop in Shannon diversity in response to amoxicillin treatment in control iron groups, and this trend does not change between introduction of different sources and concentrations of iron, except the introduction of + 75 μM FeCl_3_, which causes a reduction in the base Shannon diversity compared to the control iron group without amoxicillin, and a significant drop (^#^*p* < 0.05) in diversity when 2 μg/mL amoxicillin is added (Figure 3B). The Bray–Curtis dissimilarity PCoA plot showed that samples clustered by presence or absence of amoxicillin on the PC1 axis, and no difference in clustering between iron concentrations or groups (Figure 3C). The normalized relative abundance breakdown of the treated communities can be seen in Figure 3D, with the *Bacteroides, Escherichia-Shigella, Enterococcus,* and *Parabacteroides* genera shown separately (Figures 3E,F; Supplementary Figures S3A,B).

Looking at the OD normalized relative abundance we found that in the absence of added free iron, *Bacteroides* was elevated by 2 μg/mL AMX but was fully eliminated at 10 μg/mL AMX. Iron supplementation in both free forms led to an elimination of *Bacteroides* at the protected low AMX concertation. This is a direct contradiction to the protection we observed with hemin, where further increases in hemin lead to increased amoxicillin protection. Percent growth curves generated for *B. fragilis* and *B. thetaiotaomicron* show that there is no change in MIC_90_ values for either species when grown in different concentrations and types of free iron (Supplementary Figures S3C,D), indicating that iron in these strains does not increase amoxicillin susceptibility in the presence of different free iron sources. It should again be noted that the species identified within these samples contains mostly an unidentified species, and the unique effect hemin has as well as the lack of protection for free iron may be specific to this strain.

Similarly, *Escherichia-Shigella* did not show the same level of protection with free iron (Figures 3D,F) as with hemin (Figures 2D,F). We did observe that a higher concentration of FeCl_3_ limits the growth of *Escherichia-Shigella* when grown without amoxicillin (Figure 3F), though amoxicillin susceptibility did not significantly change because of different forms of iron. The MIC_90_ of *E. coli* Nissle however does not change when given any type or concentration of iron (Supplementary Figure S3E). This contradicts what was seen with hemin, where additions of hemin increased the MIC_90_ at least 10-fold. This indicates that *E. coli* is particularly adapted to using hemin to resist amoxicillin killing and not matching concentrations of free ferric or ferrous iron.

*Parabacteroides* do not grow well with the addition of any free form of iron, regardless of amoxicillin introduction (Supplementary Figure S3A), which is contrary to the result seen with hemin in Figure 3D and Supplementary Figure S3G where the bacteria was protected by supplementation and bloomed with hemin. The *Enterococcus* genus was also isolated and monitored. The addition of amoxicillin in media with no iron supplementation cause a stepwise reduction of *Enterococcus* (Supplementary Figure S3B). The genus also experienced a slight bloom in response to a small amount of amoxicillin when given FeSO_4_ but not when given FeCl_3_ and experienced a drop afterwards with large amounts of amoxicillin. The MIC_90_ for *E. faecalis* showed no changes in response to the introduction of different iron supplementations (Supplementary Figure S3F). This contradicts the results seen in Supplementary Figure S1F, where supplementation of hemin immediately potentiated the susceptibility of amoxicillin. This strain is not the dominant strain or species identified in our dataset, and it should be noted that this may be unique to *E. faecalis* UWH 1921.

## Discussion

In this study, we aimed to (1): create an *ex vivo* pipeline that uses *ex vivo* culturing techniques to allow for the study of the relationship between antibiotics and metabolic modulators on complex microbial makeup, and (2): deploy this pipeline to understand the relationship between different form of iron and amoxicillin susceptibility on complex microbial communities. Approximately 25% of individuals worldwide suffer from some form of anemia, with a majority of cases caused by iron deficiency (Hess et al., 2023), so studying the effect antibiotics have on individuals requiring iron supplementation is important for global human health. In its current state, this pipeline can be used to test the effects of metabolic modulators on amoxicillin susceptibility and can be used to gain real-time information about community growth. These samples are stable for 24 h and can retain 77% of total species, though further culturing times can reduce retention.

Treatment of microbial communities with amoxicillin mirrored results seen *in vivo*, where introduction of antibiotics caused a bloom of Bacteroidetes and Proteobacteria (Cabral et al., 2019; Penumutchu et al., 2023; Cuisiniere et al., 2021). These blooms can likely be attributed to a combination of intrinsic resistances and metabolically induced tolerance. When cultures were treated with various forms of iron, hemin was shown to have the most drastic effect on the communities’ growth and community makeup when combined with amoxicillin. Treatment of communities that were co-supplemented with hemin with higher amounts of amoxicillin displayed increased overall growth that rivaled OD_600_ values of untreated, unsupplemented cultures after 24 h. These communities were dominated by four particular genera when treated with a combination of hemin and amoxicillin: *Bacteroides, Escherichia-Shigella, Parabacteroides,* and *Parasutterella*. These genera were protected against amoxicillin killing in the presence of hemin. In all experiments, hemin was added at time 0 h. A mid-course addition was not tested, though given that iron availability can be growth-phase dependent, assessing hemin addition mid-exponential phase is a worthwhile follow-up. In addition, we tested the impact of hemin, but not heme; however, it is possible that some reduction of one molecule to the other may have occurred as a result of bacterial activity. The increase in *Bacteroides* and *Parabacteroides* due to iron and antibiotic combination effects has been previously reported in murine models, though this increase was reported due to the addition of iron (II) sulfate to murine diets, rather than hemin (Cuisiniere et al., 2021). We also note that excess hemin can directly enhance the growth of select taxa, even in the absence of antibiotic, which may shift competitive dynamics to suppress non-hemin-utilizing competitors (Meslé Margaux et al., 2023).

*Bacteroides* are abundant in the gut microbiome (Wexler, 2007; Zafar and Saier, 2021; Comstock and Coyne, 2003), and major shifts in *Bacteroides* abundances can lead to dysbiosis and issues to health. The genus also contains many opportunistic pathogens and has been isolated from infections across the body (Wexler, 2007; Zafar and Saier, 2021; Brook, 2016). *Bacteroides* are capable of sequestering iron and harnessing the use of siderophores produced by not only themselves, but other bacteria, in order to obtain iron (Spiga et al., 2023; Rocha et al., 2019). In particular, *B. vulgatus* can avoid spending energy on iron retrieval resources through using siderophores produced from other species (Hibbing et al., 2010). *B. vulgatus* also has been shown to employ a specific ferrous iron transporter FeoAB to resist metronidazole through the import of iron molecules (Veeranagouda et al., 2014). Heme molecules have been shown to further support the growth of *B. thetaiotaomicron*, though this had no effect on overall MIC_90_ values in our data *in vitro* (Meslé Margaux et al., 2023). The *Parasutterella* genus blooms in the presence of higher concentrations of amoxicillin when the media is supplemented with higher concentrations of hemin. *Parasutterella* are considered key members of the GI tract microbiome in humans (Ju et al., 2019), and major increases in *Parasutterella* levels have been implicated in various disease states such as Crohn’s Disease and obesity (Chiodini et al., 2015; Blasco-Baque et al., 2017; Chen et al., 2018). In previously reported data, the genus *Parasutterella* and *P. excrementihominis* both increase in high iron environments when treated with amoxicillin, though this is due to free iron (Cuisiniere et al., 2021). The genus *Parabacteroides* also blooms in the presence of amoxicillin when supplemented with high concentrations of hemin. The *Parabacteroides* genus is also a major component of the healthy gut microbiome (Sóki et al., 2024), much like *Bacteroides*. The *Escherichia-Shigella* genus also exhibited a shift in amoxicillin susceptibility with increasing hemin concentrations and increased the MIC_90_ of *E. coli* Nissle 10-fold *in vitro*. The addition of much higher concentrations of FeCl_3_, however, has been shown to have no effect on the MLC of *E. coli* (Choi et al., 2022), hinting that this effect is unique to hemin. While some *E. coli* and *Shigella* strains are key food-borne pathogens, others tend to be relatively harmless or beneficial to the microbiome’s homeostasis (Singha et al., 2023; Zaidi and Estrada-García, 2014; Scaldaferri et al., 2016). Some studies have shown that high iron supports the growth of *E. coli in vitro* and *in vivo* (Michels et al., 2019; Telang et al., 2001). Other studies used iron chelators aerobically to show that reductions in iron reduced ampicillin killing, another antibiotic of the penicillin class (Kohanski et al., 2007). The difference in observed results may be explained by different forms of iron used or that our work was performed anaerobically where the production of hydroxyl radicals from the Fenton reaction is less applicable.

Free iron supplementation into the media on the other hand did not have as drastic an effect on amoxicillin susceptibility. Overall, the change in optical density over time was not affected by the presence of any type or concentration of iron that was matched in concentration to that of hemin previously mentioned. Free iron also did not significantly alter the diversity of these samples. The presence of 75 μM FeCl_3_ prevented growth of *Bacteroides*, *Escherichia-Shigella*, *Parabacteroides*, and *Parasutterella*. The phenomenon we observed with high concentrations of hemin on amoxicillin susceptibility was not present when treated with free ferric iron. It should be noted that, though murine models have shown increases in *Bacteroides* and *Parasutterella* when given free iron and antibiotics, these mice are given substantially higher concentrations of dietary FeSO_4_ (500 mg/kg) and are subject to oral metronidazole and neomycin treatment rather than amoxicillin (Cuisiniere et al., 2021). The genus *Clostridium sensu stricto 1*, however, does seem to experience an increase in antibiotic resistance to amoxicillin as a result to supplementation of the media with free Fe (III), though this was not significant through MaAsLin2. This genus contains a large number of both pathogens like *C. botulinum and C. tetani*, as well as beneficial species such as *C. butyricum* (Li et al., 2023; Peck and van Vliet, 2016; Garrigues et al., 2022; Cassir et al., 2016). Some strains of *C. butyricum* have been identified as pathogenic, however, through their ability to produce toxins like the botulinum neurotoxin (Fenicia et al., 2007). The genus *Bifidobacterium* also grows in the presence of free Fe (II) and further increases of iron sulfate allow for a shift in amoxicillin susceptibility; *Bifidobacterium* blooms in the presence of 2 μg/mL amoxicillin when treated with 75 μM FeSO_4._ Many *Bifidobacterium* species have the capacity to retain iron due to sufficient iron sequestration mechanisms (Vazquez-Gutierrez et al., 2016; Vazquez-Gutierrez et al., 2017). In our dataset, these species were identified as *B. longum, B. kashiwanohense, B. adolescentis,* and *B. breve*. Overall, with the exception of 75 μM FeCl_3_, we saw quite similar results with free ferric and ferrous iron, likely explained through the reduction of Fe^3+^ to Fe^2+^ anaerobically (Su et al., 2020). Quantitative differences did not show significance when probed using MaAsLin2 to identify the combination effects between amoxicillin and free iron.

The bacteria that were protected against amoxicillin killing through the supplementation of hemin into the media are all robust regulators of iron entering and exiting the cell; thus, they are much more likely to maintain iron at optimal levels with and without supplementation. However, during supplementation they are uniquely able to use a variety of techniques for sequestering iron from their environment. Siderophores produced by these bacteria are also much more efficient at removing iron from cofactors such as hemin than obtaining free iron (Gräff and Barry, 2024; Smith and Wilks, 2012; Contreras et al., 2014). *E. coli* strains have been shown to produce a variety of iron uptake systems, and more virulent strains are shown to produce more siderophores (Cavas and Kirkiz, 2022; Gao et al., 2012). The key question brought up by our work is: why doesn’t hemin induce susceptibility as expected based on the role of iron in elevated metabolism? One key aspect is the anaerobic nature of our experiment where the added toxicity of iron through oxidative damage may not play a role in antimicrobial potentiation. In fact, a higher abundance of iron may support lower levels of metabolic activity required for damage and stress responses. An additional explanation is that the surviving species may have intrinsic beta-lactam resistance mechanisms that are either supported by or exaggerated by iron supplementation. It is also plausible that these cells are allocating resources toward iron regulation and uptake that may otherwise be used in futile cycling processes that would potentiate amoxicillin susceptibility. Future transcriptional profiling could probe this possibility. Analysis of markers of antimicrobial response such as the SOS response or other general stress responses (Nilson et al., 2024; Courcelle and Hanawalt, 2003; Cory et al., 2024) in combination with signatures of metabolic activity would elucidate the role iron has on metabolism and amoxicillin susceptibility (Nilson et al., 2024). For example, if iron availability upregulates metabolism while amoxicillin does not elicit a stress response, this would indicate that iron-based protection is acting through a resistance-growth enhancement direction. On the other hand, if iron availability reduces metabolic activity while the antibiotics do indeed trigger a stress response, this would indicate that iron leads to decreased sensitivity through reduction of metabolic activity. Finally, it is also possible that both metabolism and the stress responses are upregulated at the same time, likely indicating a protection mediated by an elevated capacity to deal with cellular damage.

## Conclusion

The global rise of antibiotic-resistant infections underscores the urgent need for new strategies to enhance antibiotic efficacy, particularly within complex microbial ecosystems like the human gut. Such strategies can be developed and systematically tested using *ex vivo* systems that preserve the structure and diversity of native microbial communities while enabling controlled, high-throughput experimentation. The use of iron to potentiate amoxicillin susceptibility has seen promise as a combinational therapeutic approach to eliminate bacteria naturally resistant to amoxicillin. In this study, we employed a unique *ex vivo* culturing approach to monitor the effects of iron on amoxicillin susceptibility. This pipeline allows for a high level of consistency in community inputs for an apples-to-apples comparison in susceptibility. This study showed that, contrary to expectation, higher concentrations of ferric iron trapped in a cofactor (hemin) caused communities to bloom when they were treated with amoxicillin. These communities were dominated by genera in the phyla Bacteroidetes and Proteobacteria: *Bacteroides*, *Escherichia-Shigella*, *Parabacteroides*, and *Parasutterella*. This protection was not seen, however, when matched concentrations of free iron were added to the communities and treated with amoxicillin. These findings suggest that the form of iron—not just its presence—can critically shape microbial community resilience under antibiotic stress, likely through modulation of species-specific metabolic pathways and stress responses.

Given the widespread use of both antibiotics and iron supplements, especially in vulnerable populations such as those with iron-deficiency anemia, these results have important clinical implications. Understanding how metabolic context influences antibiotic efficacy may help optimize therapeutic regimens and minimize off-target impacts on the microbiome. Furthermore, our *ex vivo* pipeline offers a powerful platform to test a wide range of metabolic or dietary interventions in combination with antimicrobials. Future work employing transcriptomics, metabolomics, and ultimately *in vivo* validation will be essential to uncover the mechanisms underlying hemin-mediated protection and determine whether these effects can be harnessed—or mitigated—to improve clinical outcomes.

## Limitations

While this study demonstrates the utility of *ex vivo* systems in profiling metabolic modulation of antibiotic susceptibility, several limitations should be noted. Host factors are absent in this model, and media composition may bias community growth. Although community structure was largely preserved in the short time frame, extended culturing reduced species retention. Only one human-derived microbiome was tested for the effects of iron on microbial susceptibility to amoxicillin, which enabled experimental consistency but limited generalizability across diverse hosts. Some taxa remained unclassified at the species level, and antibiotic susceptibility was validated only for selected isolates with amoxicillin alone. Lastly, the protective effects of hemin remain mechanistically unresolved, requiring further functional validation, and effects may vary with different beta-lactams or other classes of antibiotic.

## Data Availability

The original contributions presented in the study are publicly available. This data can be found in the NCBI BioProject database under BioProject Accession PRJNA1269992.
