# Supplementary material for: Differential impacts of hemin and free iron on amoxicillin susceptibility in ex vivo gut microbial communities
Source: Front Microbiol. 2025 Dec 1;16:1629464. doi: 10.3389/fmicb.2025.1629464 (PMC12702856; doi:10.3389/fmicb.2025.1629464)

**Supplementary Figure Legends**

**Figure S1: Validation of community culturing pipeline shows successful species retention over a 48-hour period. (A)** Alpha diversity of clinical patient derived human stool sample cultures as measured by Shannon diversity index. Black lines in the box & whisker plot indicate the mean, with whiskers identifying minimum and maximums of the data for n = 1-16. **(B)** PCoA of beta diversity of samples via Bray-Curtis Dissimilarity. **(C)** Average relative abundance of bacteria families of culture samples over 48 hours. Data are represented as average relative abundance ± STD for n = 1-8. **(D)** Percent species still present after culturing samples over 24 and 48 hours. Data are represented as the number of species that retain at least 1% of their input community’s makeup divided by the total number of species identified in the community makeup and presented as an average percent.

**Figure S2: Relationship between hemin and amoxicillin susceptibility in some genera and effect of hemin on amoxicillin MIC_90_ in key species.** **(A)** Average normalized relative abundance of *Parasutterella* genus extrapolated from Figure 3D. Data are represented as average relative abundance normalized against highest OD_600_ of experiment ± STD for n = 4. **(B)** Average normalized relative abundance of *Enterococcus* genus extrapolated from Figure 3D. Data are represented as average relative abundance normalized against highest OD_600_ of experiment ± STD for n = 4. **(C)** Percent growth curve of *B. fragilis* treated with amoxicillin and hemin. MIC_90_ calculated using Gompertz equation for MIC determination where % growth = 10%. Data represented as average percent growth ± STD for n = 3-6. **(D)** Percent growth curve of *B. thetaiotaomicron* treated with amoxicillin and hemin. MIC_90_ calculated using Gompertz equation for MIC determination where % growth = 10%. Data represented as average percent growth ± STD for n = 3-6. **(E)** Percent growth curve of *E. faecalis* treated with amoxicillin and hemin. MIC_90_ calculated using Gompertz equation for MIC determination where % growth = 10%. Data represented as average percent growth ± STD for n = 3.

**Figure S3: Relationship between free iron and amoxicillin susceptibility in some genera and effects of free iron on amoxicillin MIC_90_ in key species. (A)** Average normalized relative abundance of *Parabacteroides* genus extrapolated from Figure 4D. Data are represented as average relative abundance normalized against highest OD_600_ of experiment ± STD for n = 4. **(B)** Average normalized relative abundance of *Enterococcus* genus extrapolated from Figure 4D. Data are represented as average relative abundance normalized against highest OD_600_ of experiment ± STD for n = 4. **(C)** Percent growth curve of *B. fragilis* treated with amoxicillin and free iron. MIC_90_ calculated using Gompertz equation for MIC determination where % growth = 10%. Data represented as average percent growth ± STD for n = 3.  **(D)** Percent growth curve of *B. thetaiotaomicron* treated with amoxicillin and free iron. MIC_90_ calculated using Gompertz equation for MIC determination where % growth = 10%. Data represented as average percent growth ± STD for n = 3. **(E)** Percent growth curve of *E. coli Nissle* treated with amoxicillin and free iron. MIC_90_ calculated using Gompertz equation for MIC determination where % growth = 10%. Data represented as average percent growth ± STD for n = 3. **(F)** Percent growth curve of *E. faecalis* treated with amoxicillin and free iron. MIC_90_ calculated using Gompertz equation for MIC determination where % growth = 10%. Data represented as average percent growth ± STD for n = 3.

**Supplementary Figures**

**Figure S1:** Validation of community culturing pipeline shows successful species retention over a 48-hour period.*
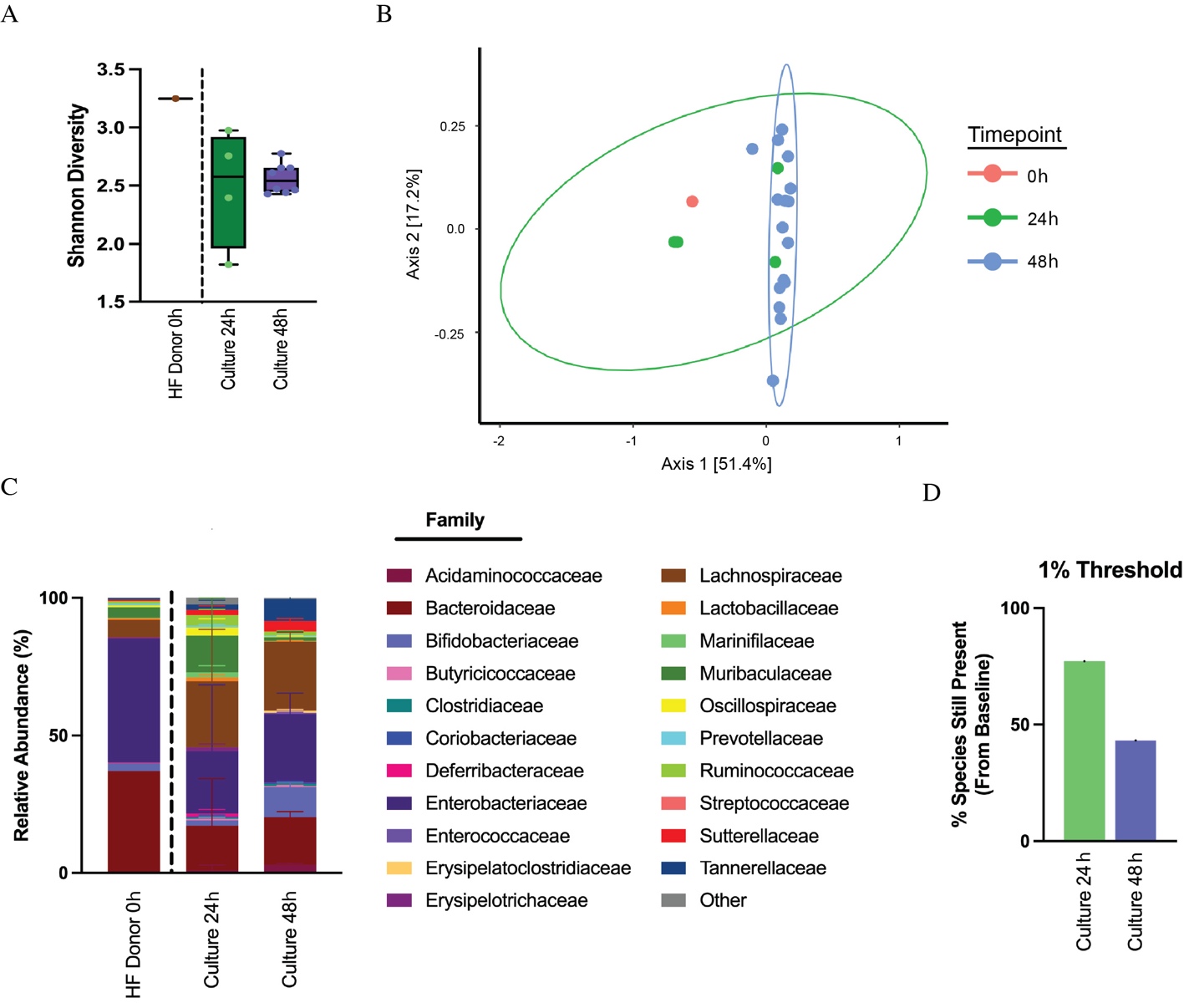
*

**Figure S2:** Relationship between hemin and amoxicillin susceptibility in some genera and effect of hemin on amoxicillin MIC_90_ in key species.
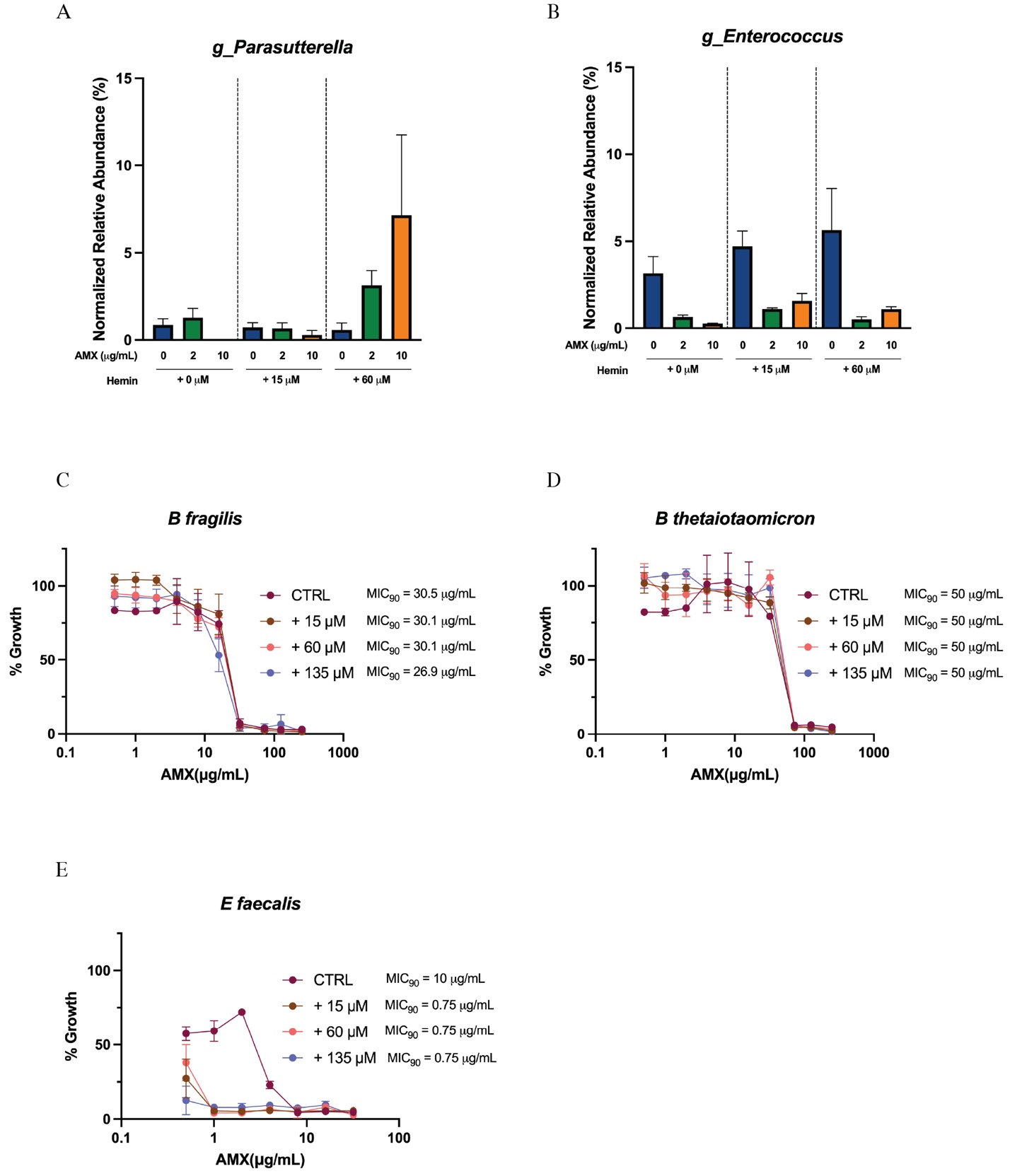


**Figure S3:** Relationship between free iron and amoxicillin susceptibility in some genera and effects of free iron on amoxicillin MIC_90_ in key species.


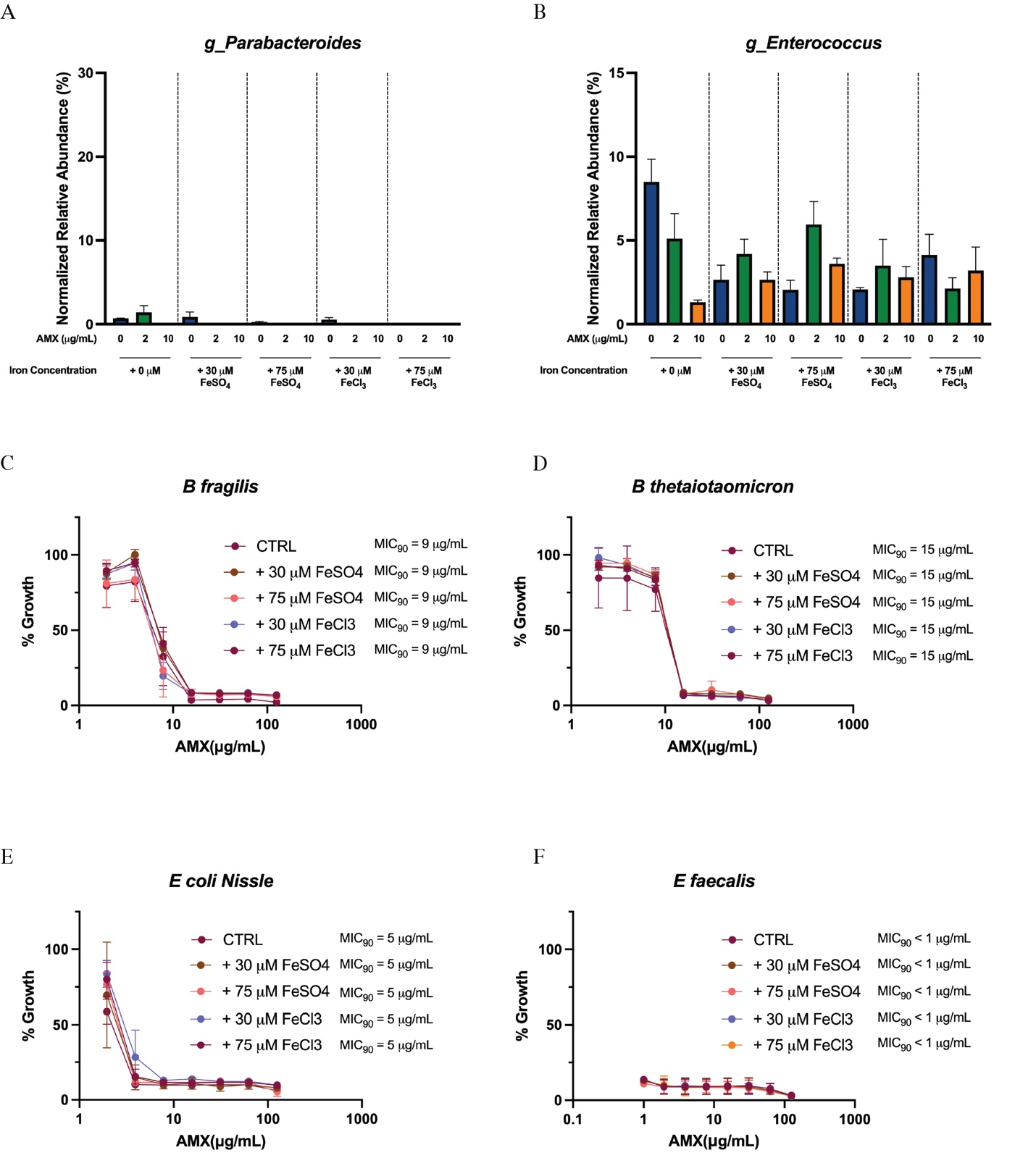

Supplement: Supplementary file 1 [file Table_1.DOCX]
